# Nonenhanced MR angiography of the pulmonary arteries using single-shot radial quiescent-interval slice-selective (QISS): a technical feasibility study

**DOI:** 10.1186/s12968-017-0365-3

**Published:** 2017-06-30

**Authors:** Robert R. Edelman, Robert I. Silvers, Kiran H. Thakrar, Mark D. Metzl, Jose Nazari, Shivraman Giri, Ioannis Koktzoglou

**Affiliations:** 1 Department of Radiology, NorthShore University HealthSystem, 2650 Ridge Avenue, Evanston, IL 60201 USA; 2 Feinberg School of Medicine, Northwestern University, Chicago, USA; 3 The University of Chicago Pritzker School of Medicine, Chicago, USA; 4 Siemens Medical Solutions USA, Inc., Chicago, USA

**Keywords:** Radial, Quiescent-interval slice-selective, Breath-holding, Navigator-gated, Cardiac

## Abstract

**Background:**

For evaluation of the pulmonary arteries in patients suspected of pulmonary embolism, CT angiography (CTA) is the first-line imaging test with contrast-enhanced MR angiography (CEMRA) a potential alternative. Disadvantages of CTA include exposure to ionizing radiation and an iodinated contrast agent, while CEMRA is sensitive to respiratory motion and requires a gadolinium-based contrast agent. The primary goal of our technical feasibility study was to evaluate pulmonary arterial conspicuity using breath-hold and free-breathing implementations of a recently-developed nonenhanced approach, single-shot radial quiescent-interval slice-selective (QISS) MRA.

**Methods:**

Breath-hold and free-breathing, navigator-gated versions of radial QISS MRA were evaluated at 1.5 Tesla in three healthy subjects and 11 patients without pulmonary embolism or arterial occlusion by CTA. Images were scored by three readers for conspicuity of the pulmonary arteries through the level of the segmental branches. In addition, one patient with pulmonary embolism was imaged.

**Results:**

Scan time for a 54-slice acquisition spanning the pulmonary arteries was less than 2 minutes for breath-hold QISS, and less than 3.4 min using free-breathing QISS. Pulmonary artery branches through the segmental level were conspicuous with either approach. Free-breathing scans showed only mild blurring compared with breath-hold scans. For both readers, less than 1% of pulmonary arterial segments were rated as “not seen” for breath-hold and navigator-gated QISS, respectively. In subjects with atrial fibrillation, single-shot radial QISS consistently depicted the pulmonary artery branches, whereas navigator-gated 3D balanced steady-state free precession showed motion artifacts. In one patient with pulmonary embolism, radial QISS demonstrated central pulmonary emboli comparably to CEMRA and CTA. The thrombi were highly conspicuous on radial QISS images, but appeared subtle and were not prospectively identified on scout images acquired using a single-shot bSSFP acquisition.

**Conclusions:**

In this technical feasibility study, both breath-hold and free-breathing single-shot radial QISS MRA enabled rapid, consistent demonstration of the pulmonary arteries through the level of the segmental branches, with only minimal artifacts from respiratory motion and cardiac arrhythmias. Based on these promising initial results, further evaluation in patients with suspected pulmonary embolism appears warranted.

## Background

Patients presenting with suspected pulmonary embolism (PE) are routinely evaluated by computed tomography angiography (CTA) [1]. The risk of having a pulmonary embolism doubles for each 10 years after age 60 [https://www.nhlbi.nih.gov/health/health-topics/topics/pe/atrisk]. Given the nearly 40% prevalence of chronic kidney disease among patients over the age of 60 years [2] and the potentially nephrotoxic effects of iodinated contrast agents, there is a substantial need for a safer imaging option in this patient group. First-pass contrast-enhanced MR angiography (CEMRA) of the pulmonary arteries is a promising alternative to CTA that avoids exposure to iodinated contrast agents and ionizing radiation [3]. However, potential challenges include the requirement for breath-holding, which may be problematic for dyspneic patients, and incomplete pulmonary artery opacification due to mistiming of the data acquisition with respect to the gadolinium bolus. Moreover, gadolinium-based contrast agents are absolutely contraindicated in pregnant patients and relatively contraindicated in those with severe renal impairment [4]. For patients with contraindications to CTA or CEMRA, a nonenhanced MRA (NEMRA) alternative would be useful.

Recently, a radial quiescent-interval slice-selective (QISS) technique was described for breath-hold imaging of the coronary arteries [5]. Radial k-space trajectories provide several advantages over Cartesian trajectories including reduced motion sensitivity, more flexible control over temporal and spatial resolution, and higher undersampling factors [6, 7]. For coronary artery QISS, two or more shots are typically required to achieve sufficiently high temporal resolution, on the order of 150 msec or less, to minimize blurring from coronary motion. Such high temporal resolution is not needed for imaging of the pulmonary arteries, which allows the use of a more efficient single-shot radial QISS acquisition. Not only does the use of a single-shot acquisition at least double the number of slices that can be acquired in each breath-hold, it avoids artifacts from shot-to-shot signal variations caused by respiratory motion or cardiac arrhythmias. Given its high imaging efficiency and resistance to motion artifacts, single-shot radial QISS could have potential utility as a nonenhanced option for evaluating patients with suspected pulmonary embolism. As an initial step, we performed a technical feasibility study to evaluate pulmonary arterial conspicuity using breath-hold and free-breathing implementations of single-shot radial QISS.

## Methods

This investigational review board (IRB)-approved study was conducted on a 1.5 Tesla scanner (MAGNETOM Avanto, Siemens Healthcare, Erlangen, Germany). Two groups of subjects were studied: (1) 3 healthy subjects; and (2) 11 patients (10 male, age range 50–73 years) who were scheduled for pulmonary vein isolation due to recurrent or persistent atrial fibrillation and had recently undergone CTA for anatomical evaluation of the pulmonary veins and heart. This patient cohort additionally provided the opportunity to evaluate the robustness of the radial QISS technique with respect to cardiac arrhythmias, since six patients were in atrial fibrillation at the time of the MR exam. Retrospective approval from the IRB was obtained for one additional patient: a 68-year-old male with shortness of breath and suspected peri-valvular leak following mitral valve repair, who underwent cardiac MR which revealed clinically unsuspected central pulmonary emboli. This patient subsequently underwent a chest CTA that confirmed the MR findings.

Imaging parameters for radial QISS were developed empirically from prior studies in volunteers and patients. Typical QISS imaging parameters included: electrocardiographic gating (ECG), one slice acquired per RR interval, radial balanced steady-state free precession (bSSFP) readout with 98 views, flip angle for the bSSFP RF excitation = 120 degrees, chemical shift-selective fat suppression, in-plane inversion using a frequency offset corrected inversion (FOCI) RF pulse with thickness = 4.5 mm, TI ~ 600 msec, in-plane resolution ~1.6-mm (or 0.8-mm after interpolation), field of view ~260-mm, slice thickness ~ 3.1-mm with 30% slice overlap, number of slices ~54, equidistant azimuthal view angle increment, readout bandwidth = 1359 Hz/pixel, bSSFP repetition time (TR) ~ 2.9 msec. Breath-hold scans were acquired over 3 breath-holds (~18 slices per breath-hold) with 10 to12 seconds between breath-holds. Free-breathing radial QISS used navigator gating with a 3-mm acceptance window and both leading and trailing cross-pair navigators. In total, all 14 subjects were imaged with breath-hold QISS, while only 13 of these subjects were imaged with navigator-gated QISS. Scans were acquired in tilted coronal and axial planes.

Navigator-gated, T2-prepared fat-saturated 3D bSSFP scans were obtained using a Cartesian k-space trajectory, bSSFP RF excitation flip angle = 90 degrees, ~72 3-mm thick slices (1.5-mm after interpolation), in-plane resolution ~1–2-mm (before interpolation), 25 segments, readout bandwidth = 1313 Hz/pixel, bSSFP TR ~ 3.1 msec. Due to time constraints, navigator-gated 3D scans were only obtained in eight subjects (1 volunteer, 7 patients).

First-pass CEMRA used a breath-hold fluoro-triggered technique with scan time = 17 s, flip angle = 23 degrees, TR = 2.7 msec, TE = 1.0 msec, spatial resolution = 2-mm × 1.1-mm × 1.0-mm, ipat acceleration factor = 4, 6/8 partial Fourier in slice and phase directions. CTA was done using a standard high-pitch retrospective-ECG gated spiral protocol on a dual-source scanner (Siemens MAGNETOM Flash, Erlangen, Germany) with 0.6-mm slice thickness.

Image Analysis: Radial QISS source images and thin multi-planar reconstructions from the three healthy volunteers and 11 patients were evaluated by three radiologists, each with more than 5 years’ experience in the interpretation of pulmonary CTA and body MRA. The pulmonary arterial tree was divided into 25 segments per Jackson and Huber [8], and vessel conspicuity was rated as: 1 = vessel seen, sharp margins, negligible artifacts; 2 = vessel seen, mildly blurred margins, mild artifacts; 3 = vessel seen, markedly blurred margins, moderate artifacts; 4 = vessel not seen, severe artifacts. To determine which pulmonary artery segments were evaluable for the image analysis, CEMRA was the reference in healthy volunteers and CTA the reference in patients. Subsegmental arterial branches, as well as segments not evaluable by CEMRA (in volunteers) or CTA (in patients) were excluded from the analysis.

Statistical Analysis: Differences in image quality ratings between the three nonenhanced protocols were assessed using Friedman tests. Gwet’s AC1 was used to assess inter-reader agreement for vessels proximal to the segmental arteries, and for the segmental branches. Bonferroni-corrected *P* values <0.05 were considered statistically significant. Analyses were performed in R software (version 3.3.2, R Foundation for Statistical Computing, Vienna, Austria).

## Results

**Fig. 1:**
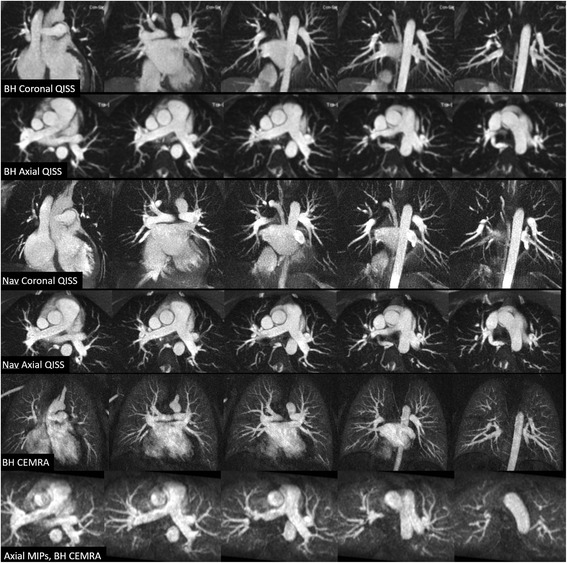
Comparison of 12-mm thick maximum intensity projections from coronal and axial breath-hold single-shot radial QISS, coronal and axial navigator-gated single-shot radial QISS, and breath-hold coronal CEMRA in a healthy subject. For radial QISS, the maximum intensity projections were reconstructed in the same orientation as the scan. For CEMRA, coronal and axial maximum intensity projections were reconstructed from a coronal scan. Both breath-hold and free-breathing QISS provided comparable depiction of pulmonary arterial anatomy to CEMRA

**Fig. 2:**
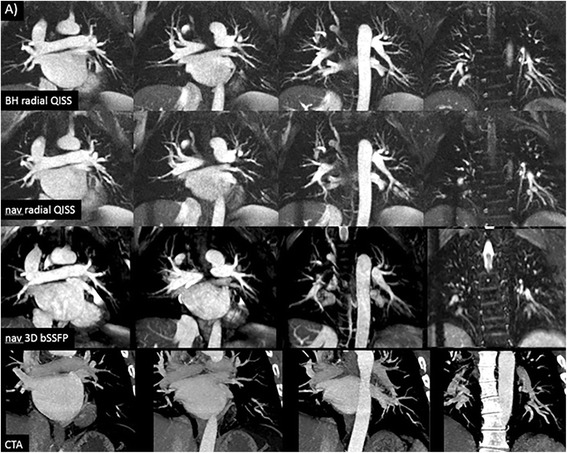
50-year-old male scheduled for pulmonary vein isolation, who was in sinus rhythm at the time of the CMR exam. **a** 12-mm thick maximum intensity projection images from coronal breath-hold radial QISS, navigator-gated radial QISS, navigator-gated 3D bSSFP, and CTA. Image quality is excellent with all MRA pulse sequences. Scan time was 3.4 min for navigator-gated QISS versus 10.3 min for navigator-gatd 3D bSSFP

**Fig. 3:**
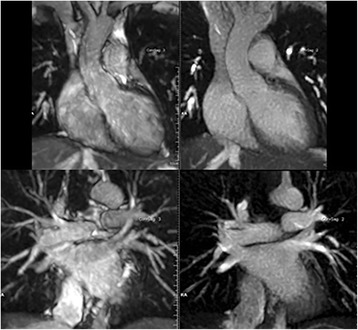
Comparison of 12-mm thick maximum intensity projections from navigator-gated 3D bSSFP (left) and navigator-gated single-shot radial QISS (right) in a healthy subject. Scans were acquired with identical spatial resolution, navigator positioning, and navigator acceptance window. Compared with 3D bSSFP, single-shot radial QISS shows better suppression of signal from pericardial fluid and less sensitivity to flow and respiratory motion artifacts, resulting in more uniform vessel signal and improved vessel sharpness

**Fig. 4:**
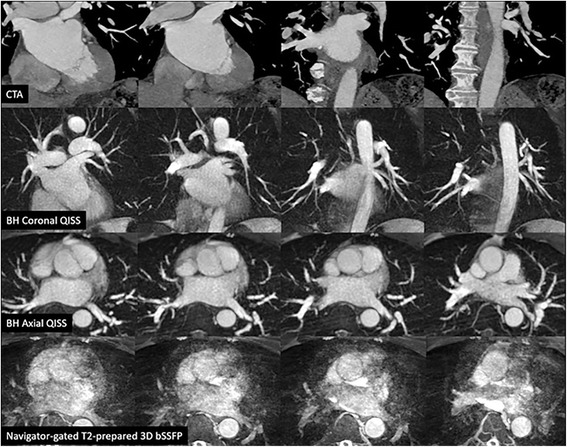
64-year-old male with poorly controlled atrial fibrillation and a rapid, variable RR interval (~480 ms) who underwent CTA and MRA prior to pulmonary vein isolation. All images are 12-mm thick maximum intensity projections. Single-shot radial QISS (*middle rows*) provided excellent depiction of the pulmonary arteries and veins in coronal and axial orientations despite the uncontrolled arrhythmia, whereas the navigator-gated 3D bSSFP images (*bottom row*) show severe artifacts

Typical breath-hold times were ~15–20 s for an 18-slice radial QISS scan, depending on the heart rate. A 54-slice, three breath-hold acquisition spanning the pulmonary arteries was completed in less than 2 min in all subjects. Scan times for a 54-slice navigator-gated QISS scan ranged from 2.0 to 3.4 min. Pulmonary artery branches were conspicuous using either breath-hold or navigator-gated radial QISS, with only mild if any blurring apparent on the free-breathing images (Figs. 1 and 2). Compared with navigator-gated 3D, vessels generally appeared sharper with navigator-gated radial QISS and there was less signal from pericardial fluid (Fig. 3). In patients with an irregular cardiac rhythm due to atrial fibrillation, the pulmonary artery branches were conspicuous on radial QISS images, but were degraded with navigator-gated 3D (Fig. 4).

Contrast-enhanced MRA in the volunteers and CTA in the patients demonstrated patency of all scored pulmonary arterial segments, with no evidence of pulmonary embolism or pulmonary artery occlusion down to the segmental level. The mean conspicuity of evaluable pulmonary arterial segments was rated in the acceptable range (1 to 3) by all three readers for both breath-hold and navigator-gated single-shot radial QISS (Table 1). Across the three readers, only 0.57% (2/350) and 0.62% (2/325) of pulmonary arterial segments were rated as “not seen” for breath-hold and navigator-gated QISS, respectively. By comparison, 31.0% (62/200) pulmonary arterial segments were rated as “not seen” during navigator-gated, T2-prepared fat-saturated 3D bSSFP. Friedman testing revealed significant differences in image quality scores between the three nonenhanced protocols (*P* < 0.05). Mean image quality scores for breath-hold QISS, navigator-gated QISS and T2-prepared fat-saturated 3D bSSFP were 2.1, 2.3 and 2.7 respectively (*P* < 0.001 across nonenhanced protocols), and 2.0 for CEMRA. In general, breath-hold QISS provided better image quality than CEMRA for the pulmonary arteries through the level of the lobar branches, while the converse was true for the segmental branches. However, the number of subjects with CEMRA was too small for statistically meaningful comparison.

**Table 1:** Image quality ratings

Segment	Nonenhanced QISS BH	Nonenhanced QISS Nav	Nonenhanced 3D Nav bSSFP	Friedman * P*-value	CEMRA
Main Pulmonary Artery	1.0	1.1	1.6	NS	1.4
Right Pulmonary Artery	1.1	1.2	1.6	NS	1.4
Left Pulmonary Artery	1.1	1.2	1.6	NS	1.4
Right Upper Lobar Artery	1.3	1.6	2.0	NS	1.6
Right Lower Lobar Artery	1.3	1.5	2.0	NS	1.7
Left Upper Lobar Artery	1.7	1.7	2.3	NS	1.4
Left Lower Lobar Artery	1.3	1.7	2.0	NS	1.4
RUL-Apical	2.5	2.7	3.0	<0.01	1.9
RUL-Anterior	2.6	2.7	3.0	NS	2.1
RUL-Posterior	2.5	2.7	3.0	<0.01	2.1
RML-Lateral	2.6	2.7	3.1	<0.01	2.1
RML-Medial	2.6	2.7	3.0	<0.01	2.1
RLL-Superior	2.6	2.7	3.0	<0.01	2.1
RLL-Medial Basal	2.5	2.7	3.0	<0.05	2.1
RLL-Anterior Basal	2.5	2.7	3.0	<0.01	2.1
RLL-Lateral Basal	2.6	2.7	3.1	<0.05	2.1
RLL-Posterior Basal	2.4	2.7	3.1	<0.01	2.1
LUL-Apicoposterior	2.4	2.7	3.0	<0.05	2.1
LUL-Anterior	2.4	2.7	3.0	<0.05	2.2
LUL-Superior Lingular	2.5	2.7	3.0	<0.01	2.4
LUL-Inferior Lingular	2.5	2.7	3.0	<0.05	2.4
LLL-Superior	2.5	2.7	3.0	<0.01	2.4
LLL-Anteromedial Basal	2.5	2.7	3.0	<0.01	2.4
LLL-Lateral Basal	2.4	2.6	3.1	<0.01	2.2
LLL-Posterior Basal	2.2	2.4	3.1	<0.001	2.2
All Segments	2.1	2.3	2.7	<0.001	2.0

Values are means across the three readers. *BH* breath-hold, *Nav* navigator-gated, *bSSFP* balanced steady-state free precession, *RUL* right upper lobe, *RML* right middle lobe, *RLL* right lower lobe, *LUL* left upper lobe, *LLL* left lower lobe, *NS* not significant

For the pulmonary arteries through the level of the lobar branches, inter-reader agreement was substantial for breath-hold QISS (AC1 = 0.62, *P* < 0.001), moderate for navigator-gated QISS (AC1 = 0.47, *P* < 0.001) and T2-prepared fat-saturated 3D bSSFP (AC1 = 0.45, *P* < 0.001), and poor for CEMRA (AC1 = 0.15, *P* < 0.01). For segmental branches, inter-reader agreement was fair for breath-hold QISS (AC1 = 0.24, *P* < 0.001), navigator-gated QISS (AC1 = 0.37, *P* < 0.001), T2-prepared fat-saturated 3D bSSFP (AC1 = 0.22, *P* < 0.001), and CEMRA (AC1 = 0.25, *P* < 0.001).

**Fig. 5:**
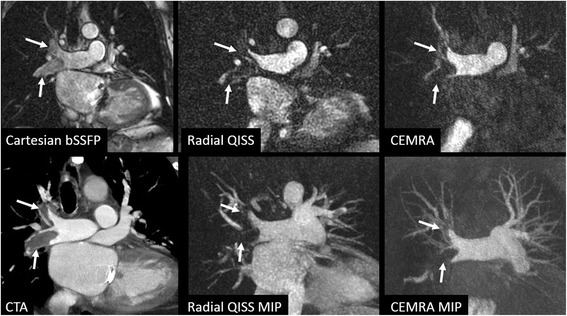
68-year-old male with shortness of breath and suspected peri-valvular leak following mitral valve repair, who underwent CMR which revealed clinically unsuspected central pulmonary emboli. *Top row*: source images from scout scan acquired using ECG-gated single-shot Cartesian bSSFP (*left*), single-shot radial QISS (*middle*), and CEMRA (*right*), *Bottom row*: multi-planar reconstruction from CTA performed immediately following the MR exam (*left*), 64-mm maximum intensity projection from radial QISS (*middle*), 64-mm maximum intensity projection from CEMRA (*right*). The pulmonary emboli are well shown by radial QISS and CEMRA. Note that the thrombi are much more conspicuous with radial QISS than with bSSFP

In one patient with clinically unsuspected pulmonary embolism, radial QISS demonstrated the central pulmonary thrombi comparably to CEMRA and CTA (Fig. 5). The thrombi were highly conspicuous on radial QISS images, but appeared subtle and were not prospectively identified on scout images acquired using an ECG-gated single-shot bSSFP acquisition.

## Discussion

Nonenhanced MRA provides a risk-free imaging alternative to CTA and CEMRA. For instance, navigator-gated 3D bSSFP is a well-described free-breathing technique that has been used to image the coronary arteries, pulmonary arteries and other great vessels in the chest [9–12]. However, no NEMRA technique has yet provided the combination of image quality, spatial resolution, scan speed, and resistance to artifacts from cardiac and respiratory motion needed to reliably evaluate the pulmonary arteries. To date, NEMRA has shown only modest sensitivity and specificity in clinical trials for pulmonary embolism [13, 14].

In this prospective technical feasibility study, breath-hold single-shot radial QISS MRA demonstrated pulmonary arterial anatomy from the main pulmonary artery through the level of the segmental branches in less than 2 min. Navigator-gated acquisitions were only slightly slower with scan times of 3.4 min or less, despite the use of both leading and trailing navigators to minimize blurring from respiration. Navigator-gated QISS tended to show slight blurring compared with breath-hold scans, but all pulmonary artery segments were adequately visualized.

In one patient, single-shot QISS clearly demonstrated multiple pulmonary emboli, whereas single-shot bSSFP showed poor conspicuity of the emboli. This case highlights that QISS, unlike bSSFP, is a highly flow-dependent imaging technique. A potential concern with QISS is saturation of in-plane flow, which is a common source of artifacts with conventional 2D time-of-flight MRA. However, we did not observe a significant degree of in-plane saturation in the pulmonary arteries through the segmental level. To maximize flow contrast, the QISS technique applies an in-plane inversion pulse prior to a quiescent interval of a few hundred milliseconds, which is then followed by a single-shot bSSFP readout. QISS differs from 2D time-of-flight MRA in that rapid systolic flow during the quiescent interval will tend to wash out saturated spins, even when the slice and vessel orientations are substantially aligned.

Cardiac arrhythmias are common in older patients due to hypertension and other co-morbidities [15]. We found that single-shot radial QISS, in contrast to navigator-gated 3D bSSFP, is resistant to motion artifacts from atrial fibrillation. The sub-second (~284 msec) readout duration for each single-shot radial QISS image effectively freezes cardiac motion and thereby avoids image artifacts. On the other hand, a navigator-gated 3D bSSFP acquisition accumulates data over dozens of cardiac cycles, so that beat-to-beat variations in cardiac dimensions and tissue signal cause image artifacts.

Compared with CEMRA, radial QISS eliminates the possibility of artifacts from imaging too early or late during the contrast infusion. The ability to repeat the scan and tailor scan planes and spatial resolution as needed, along with the capability for free-breathing acquisitions, represent other potential advantages over CEMRA, where flexibility is limited by the need to acquire all data during the first pass of contrast agent.

Further improvements in radial QISS image quality should be readily obtainable. For instance, given that these images are sparse and undersampled, iterative reconstruction techniques such as non-Cartesian SENSE or compressed sensing can be used to improve image quality [16, 17]. Simultaneous multi-slice imaging techniques can further shorten scan time, although to date most efforts have been directed to Cartesian rather than radial imaging [18].

There are several limitations to our study design. While radial QISS consistently demonstrated the pulmonary arteries down to the segmental level, imaging of smaller subsegmental branches could prove challenging due to the impact of off-resonance effects within the lung parenchyma relating to the use of a bSSFP readout. As more powerful gradient systems become available, it should be possible to shorten the bSSFP TR, thereby reducing off-resonance artifacts and improving the conspicuity of distal pulmonary arterial branches. The quality of the B0 shim is more critical with QISS, which uses a bSSFP readout, than with CEMRA, which uses a short-TE 3D spoiled gradient-echo readout. Only a small number of subjects were evaluated, and only one had significant pulmonary arterial pathology. While pulmonary emboli were anecdotally demonstrated using QISS in one patient, no assumptions can be made about the diagnostic accuracy of the technique.

## Conclusions

In summary, this technical feasibility study has demonstrated that both breath-hold and free-breathing radial QISS can rapidly and consistently depict normal pulmonary arterial anatomy down to the segmental level with an exam time on the order of a few minutes. The technique unambiguously detected central pulmonary emboli in one patient, with much improved contrast compared with standard bSSFP. Further study will be required to determine the accuracy and utility of the radial QISS technique in patients suspected of pulmonary embolism.

## Abbreviations

bSSFP: Balanced steady-state free precession
CE: Contrast enhanced
CTA: Computed tomography angiography
ECG: Electrocardiogram
MRA: Magnetic resonance angiography
PE: Pulmonary embolism
QISS: Quiescent-interval slice-selective
